# The human amniotic fluid stem cell secretome effectively counteracts doxorubicin-induced cardiotoxicity

**DOI:** 10.1038/srep29994

**Published:** 2016-07-21

**Authors:** Edoardo Lazzarini, Carolina Balbi, Paola Altieri, Ulrich Pfeffer, Elisa Gambini, Marco Canepa, Luigi Varesio, Maria Carla Bosco, Domenico Coviello, Giulio Pompilio, Claudio Brunelli, Ranieri Cancedda, Pietro Ameri, Sveva Bollini

**Affiliations:** 1 Cardiovascular Biology Laboratory, Department of Internal Medicine, University of Genova and IRCCS AOU San Martino-IST, Istituto Nazionale per la Ricerca sul Cancro, Genova, Italy; 2 Regenerative Medicine Laboratory, Department of Experimental Medicine, University of Genova, Genova, Italy; 3 Molecular Pathology Unit, IRCCS AOU San Martino-IST, Istituto Nazionale per la Ricerca sul Cancro, Genova, Italy; 4 Vascular Biology and Regenerative Medicine Unit, IRCCS Centro Cardiologico Monzino, Milano, Italy; 5 Molecular Biology Laboratory, IRCCS Istituto Giannina Gaslini, Genova, Italy; 6 Human Genetics Laboratory, E.O. Ospedali Galliera, Genova, Italy

## Abstract

The anthracycline doxorubicin (Dox) is widely used in oncology, but it may cause a cardiomyopathy with bleak prognosis that cannot be effectively prevented. The secretome of human amniotic fluid-derived stem cells (hAFS) has previously been demonstrated to significantly reduce ischemic cardiac damage. Here it is shown that, following hypoxic preconditioning, hAFS conditioned medium (hAFS-CM) antagonizes senescence and apoptosis of cardiomyocytes and cardiac progenitor cells, two major features of Dox cardiotoxicity. Mechanistic studies with mouse neonatal ventricular cardiomyocytes (mNVCM) reveal that hAFS-CM inhibition of Dox-elicited senescence and apoptosis is associated with decreased DNA damage, nuclear translocation of NF-kB, and upregulation of the NF-kB controlled genes, *Il6* and *Cxcl1*, promoting mNVCM survival. Furthermore, hAFS-CM induces expression of the efflux transporter, *Abcb1b*, and Dox extrusion from mNVCM. The PI3K/Akt signaling cascade, upstream of NF-kB, is potently activated by hAFS-CM and pre-treatment with a PI3K inhibitor abrogates NF-kB accumulation into the nucleus, modulation of *Il6*, *Cxcl1* and *Abcb1b*, and prevention of Dox-initiated senescence and apoptosis in response to hAFS-CM. These results support the concept that hAFS are a valuable source of cardioprotective factors and lay the foundations for the development of a stem cell-based paracrine treatment of chemotherapy-related cardiotoxicity.

Anthracyclines, especially doxorubicin (Dox), are the mainstay of treatment for several types of cancer. Their main side effect is cardiotoxicity, which most often presents as left ventricular dysfunction or heart failure with delayed onset^1^,^2^. In fact, many years can elapse between anthracycline administration and the appearance of anthracycline-related cardiomyopathy. Nevertheless, morbidity and mortality of heart failure secondary to anthracycline exposure may impact prognosis even more than the tumour for which chemotherapy was originally given. This scenario is currently quite common for some types of cancer, such as of the breast, and it will become more frequent in the future, as survival of oncological patients steadily increases with improved efficacy of anticancer therapies. Anthracycline cardiotoxicity may be particularly relevant for paediatric patients treated for haematological or solid tumours, since cardiomyopathy may develop over a long period of time and yet cause heart failure at a relatively young age^3^,^4^. Remarkably, clinical surveillance of patients whose cancers have been cured, or are in stable remission, is often loose and cardiac disease may remain unrecognized^5^. Thus, anthracycline cardiomyopathy is an extremely relevant medical issue with insidious evolution and growing incidence.

Unfortunately, to date no effective means exists to antagonize anthracycline cardiotoxicity^6^. Beta-blockers and angiotensin-converting enzyme inhibitors, which are well-known to reduce cardiac remodelling, have been shown to confer some protection, but it may be non-specifically due to the antagonism of maladaptive neurohormonal feedbacks. In addition, these medications are not always effective, with normalization of left ventricular ejection fraction being reported in only 11% of patients facing anthracycline cardiotoxicity^7^. On the other hand, the iron chelator dexrazoxane opposes key events in the pathogenesis of anthracycline cardiac toxicity, but there are concerns that it may limit the efficacy of anticancer drugs^5^,^6^.

Against this background, new strategies to tackle anthracycline cardiotoxicity are urgently needed. Most knowledge about the pathogenesis of anthracycline-induced heart disease comes from studies with Dox. It has been demonstrated that senescence^8^ and apoptosis^9^ of cardiomyocytes and cardiac progenitor cells (CPC) underlie Dox cardiomyopathy. At the subcellular level, Dox primarily interferes with topoisomerase-IIβ and causes DNA damage, followed by a DNA damage response and a cascade of perturbations culminating in cell cycle arrest or death^10^. Therefore, the ideal intervention to prevent Dox cardiotoxicity should limit DNA damage and elicit anti-senescent and anti-apoptotic pathways in cardiomyocytes and CPC.

Here, we demonstrate that the *secretome* (i.e. the entire set of paracrine factors secreted by cells) of human amniotic fluid-derived stem cells (hAFS) has such a profile of activity. hAFS are broadly multipotent mesenchymal progenitors isolated upon c-kit expression from the heterogeneous cell population contained in leftover samples of second trimester amniotic fluid, obtained by amniocentesis for routine prenatal screening^11^. They are endowed with both high self-renewal potential and embryonic stem cell-like properties and secrete regenerative paracrine factors which have been shown to stimulate neo-arteriogenesis, recruit host progenitor cells *in vivo*, and promote tissue regeneration in different injury models^12^,^13^. It is noteworthy that hAFS conditioned medium (hAFS-CM), which contains all the bioactive molecules secreted by the cells, has already been reported to considerably reduce cardiac ischemic damage in the short time in a rat model by exerting paracrine anti-apoptotic effects on resident cardiomyocytes^14^. Moreover, harvesting of hAFS from leftover samples of amniotic fluid collected for prenatal screening does not carry any clinically unnecessary risk of harm for either the mother or the foetus, nor does it raise ethical concerns. Therefore, in this study we suggest harnessing the hAFS secretome to antagonize the cardiotoxic side effects of Dox for future paracrine therapy.

## Results

### The hAFS secretome rescues H9c2 cells from Dox-induced senescence and apoptosis

**Figure 1:**
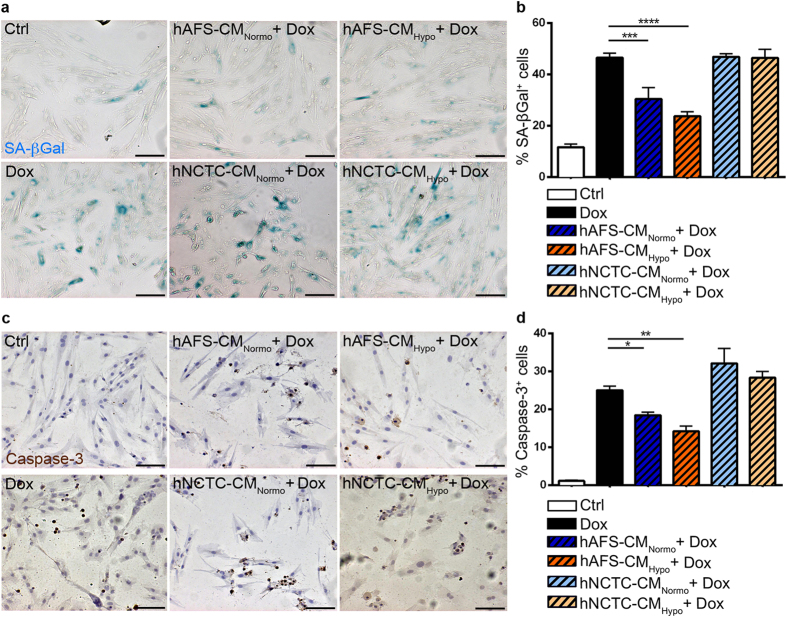
The hAFS-CM inhibits the pro-senescent and pro-apoptotic effect of Dox on H9c2 cells. (**a**) Representative images of rat H9c2 cardiomyoblasts stained for senescence associated (SA) β-galactosidase: untreated cells (*Ctrl*), cells exposed to 0.1 μM Dox (*Dox*), and cells pre-incubated with 40 μg/ml of: the hAFS-CM_Normo_ (*hAFS-CM*_*Normo*_ + *Dox*), the hAFS-CM_Hypo_ (*hAFS-CM*_*Hypo*_ + *Dox*), the hNCTC-CM_Normo_ (*hNCTC-CM*_*Normo*_ + *Dox*), or the hNCTC-CM_Hypo_ (*hNCTC-CM*_*Normo*_ + *Dox*) prior to Dox treatment. Scale bar 100 μm. (**b**) Percentage of H9c2 cells expressing SA β-galactosidase after exposure to Dox with or without pre-incubation with 40 μg/ml of the hAFS-CM or hNCTC-CM (mean ± s.e.m.). Ctrl: 11.7 ± 1.2%, Dox: 46.5 ± 1.7%, hAFS-CM_Normo_ + Dox: 30.4 ± 4.5%, hAFS-CM_Hypo_ + Dox: 23.7 ± 1.8%, hNCTC-CM_Normo_ + Dox: 46.9 ± 1.2%, and hNCTC-CM_Hypo_ + Dox: 46.4 ± 3.3%; ***p < 0.001 (p = 0.0004), ****p < 0.0001. (**c**) Representative images of H9c2 cardiomyoblasts stained for cleaved caspase-3 (Caspase-3): untreated cells (*Ctrl*), cells exposed to 1 μM Dox (*Dox*), and cells pre-incubated with 40 μg/ml of: the hAFS-CM_Normo_ (*hAFS-CM*_*Normo*_ + *Dox*), the hAFS-CM_Hypo_ (*hAFS-CM*_*Hypo*_ + *Dox*), the hNCTC-CM_Normo_ (*hNCTC-CM*_*Normo*_ + *Dox*), or the hNCTC-CM_Hypo_ (*hNCTC-CM*_*Normo*_ + *Dox*) prior to Dox treatment. Scale bar 100 μm. (**d**) Percentage of H9c2 cells expressing cleaved caspase-3 (% Caspase-3^+^ cells) after exposure to Dox with or without pre-incubation with 40 μg/ml of the hAFS-CM or hNCTC-CM (mean ± s.e.m.). Ctrl: 1.1 ± 0.2%, Dox: 25.0 ± 1.1%, hAFS-CM_Normo_ + Dox: 18.5 ± 0.8%, hAFS-CM_Hypo_ + Dox: 14.2 ± 1.4%, hNCTC-CM_Normo_ + Dox: 32.0 ± 3.9%, and hNCTC-CM_Hypo_ + Dox: 28.3 ± 1.7%; *p < 0.05 (p = 0.0433), **p < 0.01 (p = 0.0014).

An established *in vitro* model of Dox cardiotoxicity^15^,^16^ was initially used to screen the effects of the hAFS-CM. H9c2 rat embryonic cardiomyoblasts were exposed to Dox with or without prior incubation with the hAFS-CM obtained by culturing hAFS under normoxic (20% O_2_) or hypoxic (1% O_2_) conditions (hAFS-CM_Normo_ and hAFS-CM_Hypo_, respectively). Since we previously observed that hAFS are activated by the ischemic environment and promote cardiomyocyte survival in a paracrine manner^14^, hypoxic preconditioning was used as a working strategy to enrich hAFS-CM for cardioprotective factors. Indeed, hAFS-CM_Hypo_ showed an almost two-fold increase in protein concentration compared to hAFS-CM_Normo_ (485.1 ± 67.1 μg/10^6^ cells versus 266.6 ± 35.2 μg/10^6^ cells, respectively; see Supplementary Fig. S1). As shown in Fig. 1, the percentage of cells stained for senescence associated (SA) β-galactosidase^16^ (Fig. 1a,b) and cleaved caspase-3 (Fig. 1c,d) dramatically rose with Dox to values similar to those found previously^15^,^16^ and was significantly reduced by 35% and 26%, respectively, by pre-treatment with hAFS-CM_Normo_ (46.5 ± 1.7% vs 30.4 ± 4.5% of SA β-galactosidase positive cells and 25.0 ± 1.0% vs 18.5 ± 0.8% cleaved caspase-3 positive cells). The hAFS-CM_Hypo_ was even more protective, the number of senescent and apoptotic cells being decreased by 49% and 43% respectively (46.5 ± 1.7% vs 23.7 ± 1.8% of β-galactosidase positive cells and 25.0 ± 1.0% vs 14.2 ± 1.4% cleaved caspase-3 positive cells).

By contrast, Dox-elicited senescence and apoptosis were not reduced by pre-incubation with the conditioned medium from the human keratinocyte cell line, NCTC 2544 (hNCTC-CM_Normo_ and hNCTC-CM_Hypo_), used as an internal control (Fig. 1b,c). Compared with untreated cells, no significant change in SA β-galactosidase or cleaved caspase-3 expression was seen when incubating H9c2 cardiomyoblasts with the hAFS-CM_Normo_ or hAFS-CM_Hypo_ alone (see Supplementary Fig. S2).

### hAFS-CM_Hypo_ effectively protects mouse neonatal cardiomyocytes against Dox

**Figure 2:**
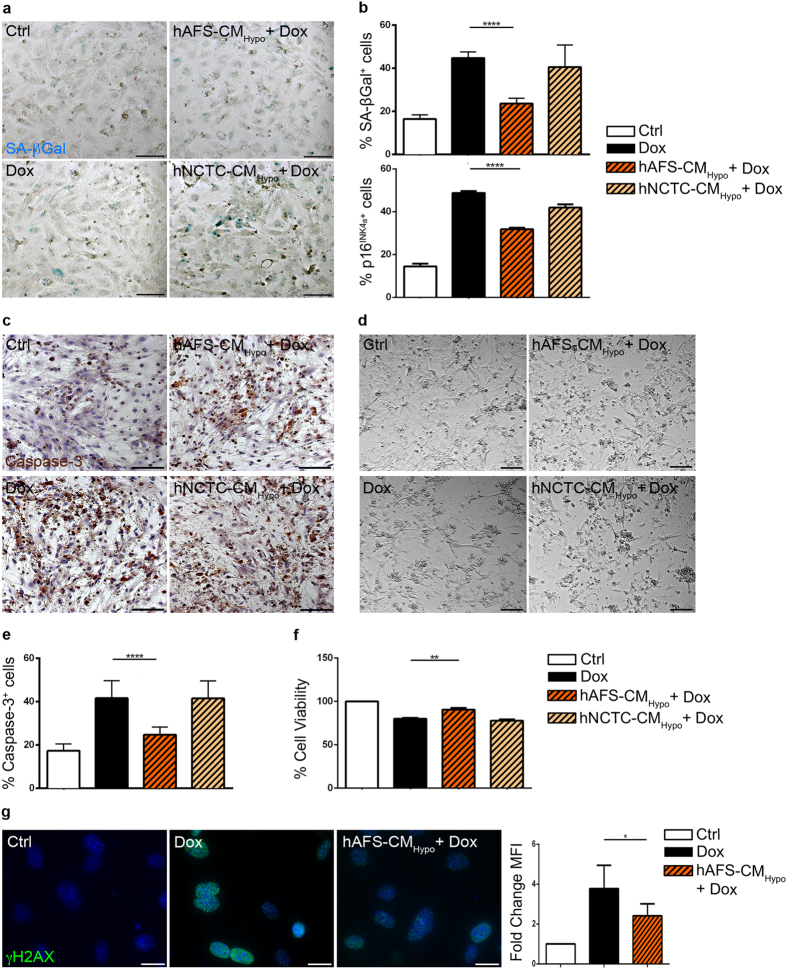
The hAFS-CM_Hypo_ is endowed with paracrine cardioprotective properties against Dox damage. (**a**) Representative images of mNVCM stained for senescence associated (SA) β-galactosidase: untreated cells (*Ctrl*), cells exposed to 0.2 μM Dox (*Dox*), and cells pre-incubated with 40 μg/ml of hAFS-CM_Hypo_ (*hAFS-CM*_*Hypo*_ + *Dox*) or hNCTC-CM_Hypo_ (*hNCTC-CM*_*Normo*_ + *Dox*). Scale bar 100 μm. (**b**) Upper panel: mNVCM expressing SA β-galactosidase after Dox exposure with or without pre-incubation with 40 μg/ml of the hAFS-CM_Hypo_ or the hNCTC-CM_Hypo_ (mean ± s.e.m.). Ctrl: 16.4 ± 1.9%, Dox: 44.7 ± 2.9%, hAFS-CM_Hypo_ + Dox: 23.6 ± 2.4%, and hNCTC-CM_Hypo_ + Dox: 40.5 ± 10.2%; ****p < 0.0001. Lower panel: mNVCM expressing p16^INK4a^ after Dox treatment with or without pre-incubation with 40 μg/ml of the hAFS-CM_Hypo_ or the hNCTC-CM_Hypo_ (mean ± s.e.m.). Ctrl: 14.5 ± 2.9%, Dox: 48.8 ± 1.8%, hAFS-CM_Hypo_ + Dox: 31.8 ± 1.5%, and hNCTC-CM_Hypo_ + Dox: 41.9 ± 3.0%; ****p < 0.0001. (**c**) Representative images of mNVCM stained for cleaved caspase-3 (Caspase-3): untreated cells (*Ctrl*), cells exposed to 1 μM Dox (*Dox*), and cells pre-incubated with the hAFS-CM_Hypo_ (*hAFS-CM*_*Hypo*_ + *Dox*) or the hNCTC-CM_Hypo_ (*hNCTC-CM*_*Normo*_ + *Dox*). Scale bar 100 μm. (**d**) Representative images of viable mNVCM by MTT assay: untreated cells (*Ctrl*), cells exposed to 1 μM Dox (*Dox*), and cells pre-incubated with the hAFS-CM_Hypo_ (*hAFS-CM*_*Hypo*_ + *Dox*) or the hNCTC-CM_Hypo_ (*hNCTC-CM*_*Normo*_ + *Dox*) prior to Dox. Scale bar 100 μm. (**e**) mNVCM expressing cleaved-caspase-3 (% Caspase-3^+^ cells) after exposure to Dox with or without pre-incubation with 40 μg/ml of the hAFS-CM_Hypo_ or the hNCTC-CM_Hypo_ (mean ± s.e.m.). Ctrl: 17.3 ± 0.8%, Dox: 41.6 ± 2.1%, hAFS-CM_Hypo_ + Dox: 24.7 ± 1.1%, and hNCTC-CM_Hypo_ + Dox: 41.5 ± 4.1%; ****p < 0.0001. (**f)** Percentage of viable mNVCM by MTT assay, after exposure to Dox with or without pre-incubation with 40 μg/ml of the hAFS-CM_Hypo_ or hNCTC-CM_Hypo_, compared to untreated cells (mean ± s.e.m.). Ctrl: 100%, Dox: 80.0 ± 0.8%, hAFS-CM_Hypo_ + Dox: 90.5 ± 2.1%, and hNCTC-CM_Hypo_ + Dox: 77.8 ± 1.4%; **p < 0.01 (p = 0.006). (**g**) Representative images of mNVCM stained for γH2AX: untreated cells (*Ctrl*), cells exposed to 1 μM Dox (*Dox*), and cells pre-incubated with 40 μg/ml of the hAFS-CM_Hypo_ (*hAFS-CM*_*Hypo*_ + *Dox*). Scale bar 20 μm. The graph on the right shows the mean fluorescence intensity (MFI) fold change after exposure to Dox, with or without pre-incubation with the hAFS-CM_Hypo_; *p < 0.05 (p = 0.0114).

Next, the cardioprotective potential of hAFS-CM_Hypo_ - which had proved to be the most effective form of hAFS-CM to prevent Dox toxicity in the experiments with H9c2 cardiomyoblasts - was confirmed on primary mouse neonatal ventricular cardiomyocytes (mNVCM). Consistent with earlier studies^16^, SA β-galactosidase positive mNVCM were about 45% after Dox treatment. Pre-incubation with hAFS-CM_Hypo_ diminished the frequency of senescent cells by 47% (Fig. 2a,b: upper panel; 44.7 ± 2.9% vs 23.6 ± 2.4% of SA β-galactosidase positive cells). Similar results were obtained by immunostaining for p16^INK4a^, with the hAFS-CM_Hypo_ decreasing the pro-senescent effect of Dox by 35% (Fig. 2b lower panel; 48.8 ± 1.8% vs 31.8 ± 1.5% of p16^INK4a^ positive cells). Moreover, there were twice as many cleaved caspase-3 positive cells with Dox as without treatment, and this increase in apoptosis was also reduced by the hAFS-CM_Hypo_ by 41% (Fig. 2c,e: 41.6 ± 2.1% vs 24.7 ± 1.1% cleaved caspase-3 positive cells). Correspondingly, cell viability, as measured by MTT assay, was increased by 10.5% by pre-incubation with hAFS-CM_Hypo_ (Fig. 2d,f). Importantly, hNCTC-CM did not prevent Dox-initiated senescence and apoptosis of mNVCM (Fig. 2b,e,f), nor were these cell responses significantly modified by hAFS-CM_Hypo_ or hAFS-CM_Normo_ alone (see Supplementary Fig. S2).

Immunoreactivity for phosphorylated H2AX (γH2AX), a sensitive indicator of DNA double-strand breaks and damage^17^, was much more widespread and intense following exposure to Dox, than in untreated mNVCM (Fig. 2g). Mean fluorescence intensity for γH2AX was increased by about 4-fold by Dox compared to untreated cells, but limited to a 2.4 fold change, corresponding to a 37% reduction, by pre-incubation with the hAFS-CM (Fig. 2g, right panel).

### hAFS-CM_Hypo_ acts through the NFkB-controlled pro-survival genes *Il6* and *Cxcl1* and the PI3K/Akt pathway

**Figure 3:**
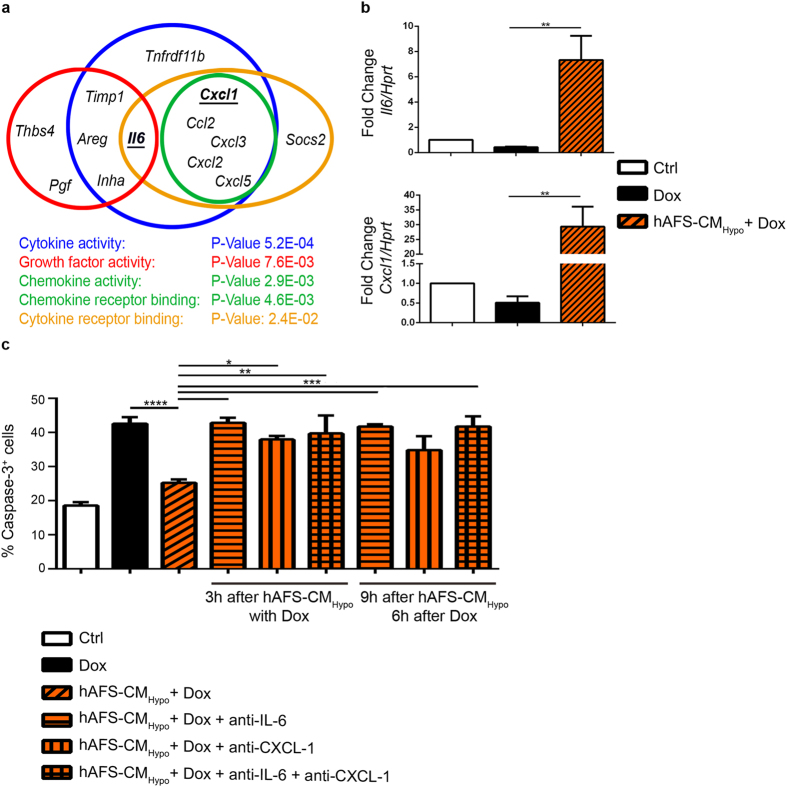
The pro-survival genes *Il6* and *Cxcl1* are up-regulated by incubation with the hAFS-CM_Hypo_ prior to Dox exposure and play a pivotal role in hAFS-CM_Hypo_ cardioprotection. (**a**) Schematic illustrating the cluster of genes coding for cytokines, chemokines, growth factors, and chemokine and cytokine receptor-binding proteins up-regulated by incubation with 40 μg/ml of hAFS-CM_Hypo_ prior to exposure to 1 μΜ Dox, according to microarray analysis. (**b**) Real time qRT-PCR showing significant up-regulation of the NF-κB-controlled pro-survival genes *Il6* and *Cxcl1* in mNVCM incubated with 40ug/ml of hAFS-CM_Hypo_ prior to treatment with 1 μM Dox; **p < 0.01 (p = 0.0015 and p = 0.0020, respectively). (**c**) Percentage of mNVCM expressing cleaved caspase-3 (% Caspase-3^+^ cells) after exposure to Dox, with or without pre-incubation with 40 μg/ml of the hAFS-CM_Hypo_ and with blocking antibodies against IL-6 (anti-IL6) and/or CXCL-1 (anti-CXCL1) added to the culture medium (mean ± s.e.m.). Ctrl: 18.55 ± 4.0%, Dox: 42.57 ± 7.7%, hAFS-CM_Hypo_ + Dox: 25.25 ± 3.5%, The following values refer to results obtained adding blocking antibodies when Dox treatment began, after 3 hours of incubation with the hAFS-CM: hAFS-CM_Hypo_ + Dox + anti-IL-6: 42.79 ± 2.7%, hAFS-CM_Hypo_ + Dox + anti-CXCL-1: 37.94 ± 1.8%, hAFS-CM_Hypo_ + Dox + anti-IL-6 and+ anti-CXCL-1: 39.68 ± 9.1%, ****p < 0.0001, ***p < 0.001 (p = 0.0002), **p < 0.01 (p = 0.0041) *p < 0.05 (p = 0.0181). The following values refer to results obtained adding blocking antibodies 6 and 9 hours after Dox treatment and hAFS-CM incubation, respectively: hAFS-CM_Hypo_ + Dox + anti-IL-6: 41.75 ± 1.1%, hAFS-CM_Hypo_ + Dox + anti-CXCL-1: 34.83 ± 7.1%, hAFS-CM_Hypo_ + Dox + anti-IL-6 + anti-CXCL-1: 41.75 ± 5.1%; ***p < 0.001 (p = 0.0006).

To identify the pathways responsible for hAFS-CM_Hypo_ antagonism of Dox cardiotoxicity, gene expression profiles were compared between mNVCM treated with Dox or incubated with hAFS-CM_Hypo_ followed by Dox exposure. Microarray analysis using Affymetrix technology revealed that pre-treatment with hAFS-CM_Hypo_ led to the up-regulation of genes coding for cytokines, chemokines, growth factors, and chemokine and cytokine receptor binding proteins (Fig. 3a and Supplementary Table S1). In particular, *Il6* and *Cxcl1* were highly expressed. Confirmatory real time qRT-PCR at 6 hours after Dox treatment demonstrated that these two genes were up-regulated by 7 and 29 fold compared to untreated cells and by 17- and 59 fold compared to Dox treatment, respectively, when the hAFS-CM_Hypo_ preceded Dox exposure (Fig. 3b). Since IL-6 and CXCL-1 have been shown to be involved in mNVCM survival^18^,^19^, we investigated whether blockade of these two cytokines might influence the protective effects of hAFS-CM_Hypo_. The decrease in apoptosis attained with hAFS-CM_Hypo_ was significantly blunted when pre-incubation with hAFS-CM_Hypo_ was followed by treatment with Dox, along with antibodies against IL-6 or IL-6 as well as CXCL-1 (either soon after or after 6 hours post Dox administration, Fig. 3c), thus indicating that hAFS-CM_Hypo_ inhibits Dox-induced apoptosis via IL-6 and, to a lesser extent, CXCL-1.

**Figure 4:**
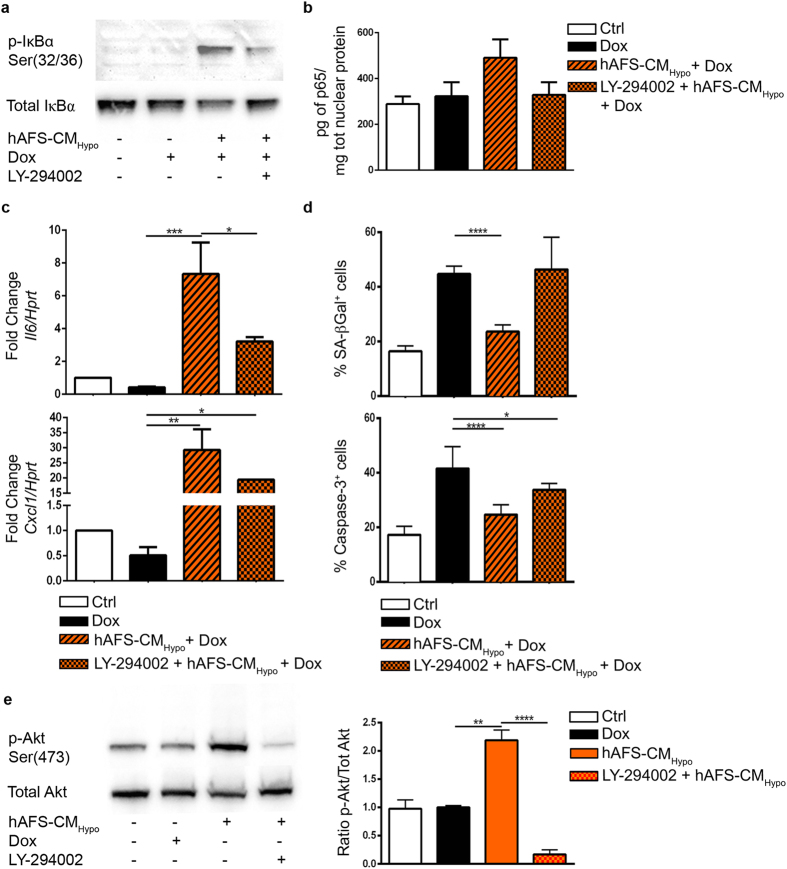
hAFS-CM_Hypo_ prevention of Dox cardiotoxicity is associated with activation of the PI3K/Akt and NF-κB pathways. (**a**) Western blot analysis for the expression of phosphorylated IκBα (Ser^32/36^ p-IκBα, 40kDa) in untreated mNVCM or mNVCM treated with 1 μM Dox, with 40 μg/ml hAFS-CM_Hypo_ prior to exposure to 1 μM Dox, or with 20 μM LY-294002 (PI3K inhibitor) followed by 40 μg/ml hAFS-CM_Hypo_ and 1 μM Dox. Full-length blots are represented in Supplementary Fig. S4. (**b**) ELISA analysis for nuclear expression of the p65 subunit of the NF-kB complex in untreated mNVCM (*Ctrl*) or mNVCM treated with 1 μM Dox (*Dox*), with 40 μg/ml hAFS-CM_Hypo_ followed by 1 μM Dox (*hAFS-CM*_*Hypo*_ + *Dox*), or with 40 μg/ml hAFS-CM_Hypo_ followed by 1 μM Dox after pre-incubation with 20 μM LY-294002 (*LY-294002* + *hAFS-CM*_*Hypo*_ + *Dox*). *Pg*: picogram; μ*g*: microgram. (**c**) Real time qRT-PCR showing that treatment of mNVCM with 20 μM LY-294002 (*LY-294002* + *hAFS-CM*_*Hypo*_ + *Dox*) also abrogated the upregulation of *Il6* and decreased that of *Cxcl1* attained by pre-incubation with 40 μg/ml hAFS-CM_Hypo_ prior to exposure to 1 μM Dox (*hAFS-CM*_*Hypo*_ + *Dox*). *p < 0.05 (p = 0.0309 and p = 0.0324, for *Il6* and *Cxcl1* respectively), **p < 0.01 (p = 0.0015), ***p < 0.001 (p = 0.0006). (**d**) Percentage of mNVCM expressing SA β-galactosidase (upper panel) and cleaved caspase-3 (% Caspase-3^+^ cells, lower panel): untreated cells (*Ctrl*), cells exposed to 1 μM Dox (*Dox*), and cells treated with 40 μg/ml hAFS-CM_Hypo_ followed by 1 μM Dox (*hAFS-CM*_*Hypo*_ + *Dox*) or with 40 μg/ml hAFS-CM_Hypo_ followed by 1 μM Dox after pre-incubation with 20 μM LY-294002 (*LY-294002* + *hAFS-CM*_*Hypo*_ + *Dox*). Note the reversion of the cardioprotective effects of the hAFS secretome with LY-294002 (LY-294002 + hAFS-CM_Hypo_ + Dox: 46.37 ± 6.84% of SA β-galactosidase positive cells and 33.80 ± 0.94% of cleaved caspase-3 positive cells, values expressed as mean ± s.e.m.; *p < 0.05 (p = 0.0208), ****p < 0.0001. (**e**) Western blot analysis (left panel) and corresponding densitometry (right panel) for phosphorylated Akt (Ser^473^ p-Akt, 60kDa) in untreated mNVCM (*Ctrl*) or mNVCM treated with 1 μM Dox (*Dox*), with 40 μg/ml hAFS-CM_Hypo_ (*hAFS-CM*_*Hypo*_), or with 20 μM LY-294002 (PI3K inhibitor) followed by 40 μg/ml hAFS-CM_Hypo_ (*LY-294002* + *hAFS-CM*_*Hypo*_). Full-length blots are represented in Supplementary Fig. S4. **p < 0.01 (p = 0.0011), ****p < 0.0001.

*Il6* and *Cxcl1* are controlled by nuclear factor kappa-light-chain-enhancer of activated B cells (NF-κB). Under basal conditions, the latter is kept inactive in the cytoplasm by inhibitor of κB alpha (IκBα). Phosphorylation of IκBα at critical serine residues leads to its degradation and relieves NF-κB, which can localize to the nucleus and modulate the transcription of target genes. Consistently, pre-incubation of mNVCM with hAFS-CM_Hypo_ before Dox treatment enhanced IκBα phosphorylation (Fig. 4a) and increased the amount of NF-κB in the nuclear compartment, as assessed by ELISA for the p65 subunit (Fig. 4b), although not to a statistically significant extent. Since the phosphoinositide 3-kinase (PI3K)/Akt cascade is a main trigger of IκBα degradation, we sought to determine whether this pathway lies upstream of the activation of NF-κB, the induction of *Il6* and *Cxcl1*, and the protection against Dox toxicity by the hAFS-CM_Hypo_. The PI3K inhibitor, LY-294002, reduced the phosphorylation of IκBα (Fig. 4a), prevented the nuclear accumulation of NF-κB (Fig. 4b), and attenuated the increase in *Il6* and *Cxcl1* (Fig. 4c). Furthermore, it abolished the inhibition of Dox-triggered senescence and apoptosis attained with the hAFS-CM_Hypo_ (Fig. 4d), indicating that hAFS-CM_Hypo_ up-regulates the NF-κB-dependent pro-survival genes, *Il6* and *Cxcl1*, and counteracts Dox cardiotoxicity at least in part via PI3K/Akt. In fact, AFS-CM_Hypo_ potently stimulated the phosphorylation of Akt twice as much compared to Dox treatment alone, an effect which was abrogated by LY-294002 (Fig. 4e).

### hAFS-CM_Hypo_ promotes Dox efflux from mouse neonatal cardiomyocytes

**Figure 5:**
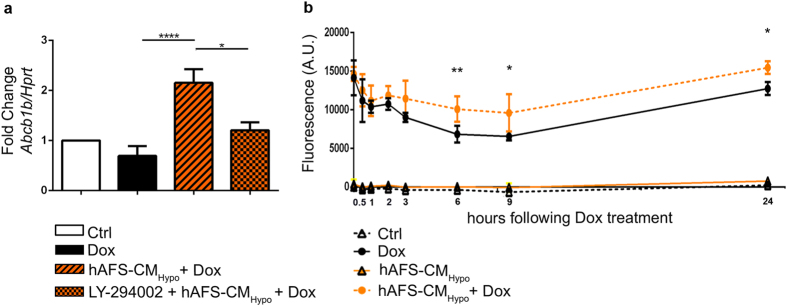
The hAFS-CM_Hypo_ induces the up-regulation of *Abcb1b* and the efflux of Dox from mNVCM. (**a**) Real time qRT-PCR showing significant up-regulation of the *Abcb1b* gene in mNVCM incubated with 40 μg/ml hAFS-CM_Hypo_ prior to exposure to 1 μM Dox (*hAFS-CM*_*Hypo*_ + *Dox*) compared to untreated mNVCM (*Ctrl*) and mNVCM exposed to Dox (*Dox*); this effect was significantly reduced when cells were treated with the PI3K inhibitor, LY-294002 (*LY-294002* + *hAFS-CM*_*Hypo*_ + *Dox)*; *p < 0.05 (p = 0.0232), ****p < 0.0001. (**b**) Quantification of Dox fluorescence in the mNVCM-conditioned medium at 0.5, 1, 2, 3, 6, 9 and 24 h following incubation with 40ug/ml hAFS-CM_Hypo_ and/or exposure to 1 μM Dox (mean ± s.e.m.). **p < 0.01 (p = 0.0058), *p < 0.05 (p = 0.0110 and p = 0.0292 for the 9 h and 24 h time point respectively). *A.U.*: Arbitrary Unit.

Since pre-incubation with the hAFS-CM_Hypo_ substantially reduced DNA damage by Dox treatment, we wondered whether the hAFS-CM_Hypo_ might also affect the retention of the drug by mNVCM besides activating protective pathways. As experimental studies have shown that the transmembrane pump ABCB1B extrudes Dox from cells and is critical to determine its toxic effects^20^, we examined the levels of this transporter in mNVCM. Pre-incubation with the hAFS-CM_Hypo_ led to a sharp increase in *Abcb1b* expression of about 3-fold and 2-fold, respectively, compared to Dox treatment alone or to untreated cells and this was prevented by LY-294002 (Fig. 5a). Since Dox is autofluorescent, the fluorescence in culture medium after treatment with the drug is a function of its efflux from cells. This feature was exploited to determine whether *Abcb1b* modulation reduced Dox accumulation in mNVCM. As shown in Fig. 5b, fluorescence in culture medium was significantly higher at 6, 9 and 24 hours after exposure to Dox, when cells were pre-incubated with hAFS-CM_Hypo_ than when they were not (Fig. 5b). Interestingly, no modulation of the multidrug resistance gene *ABCB1* nor Dox fluorescence in culture medium was observed when the human breast cancer cell line MDA-MB-231^21^, was pre-incubated with hAFS-CM_Hypo_ before being treated with Dox (Supplementary Fig. S3).

### hAFS-CM_Hypo_ antagonizes Dox toxicity on human cardiac progenitor cells

**Figure 6:**
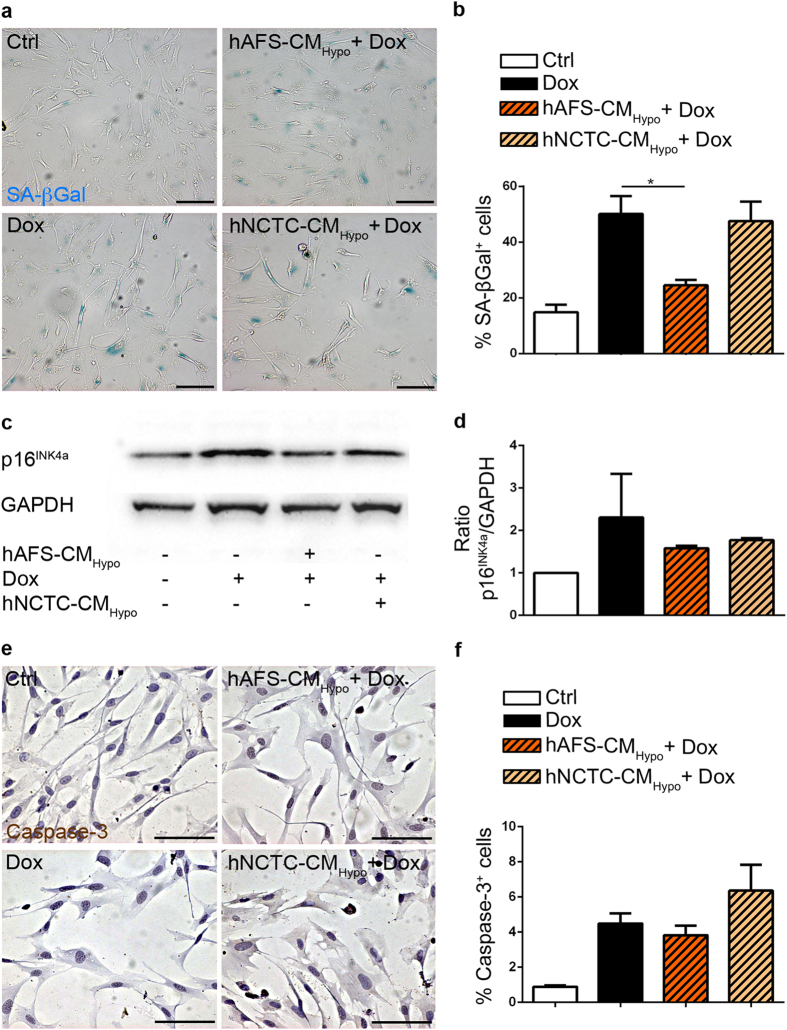
The hAFS secretome antagonizes Dox-elicited damage of human CPC. (**a)** Representative images of human c-kit^+^ CPC (hCPC) stained for senescence associated (SA) β-galactosidase: untreated cells (*Ctrl*), cells exposed to 0.2 μM Dox (*Dox*), and cells incubated with 40 μg/ml of the hAFS-CM_Hypo_ (*hAFS-CM*_*Hypo*_ + *Dox*) or with the hNCTC-CM_Hypo_ (*hNCTC-CM*_*Normo*_ + *Dox*) prior to Dox treatment. Scale bar 100 μm. (**b**) Percentage of hCPC expressing SA β-galactosidase (mean ± s.e.m.). Ctrl: 14.8 ± 2.7%, Dox: 50.1 ± 6.4%, hAFS-CM_Hypo_ + Dox: 24.6 ± 1.9%, and hNCTC-CM_Hypo_ + Dox: 47.5 ± 7.0%; *p < 0.05 (p = 0.0285). (**c**) Representative western blot for the expression of the senescence marker p16^INK4a^ (17kDa; housekeeping GAPDH, 37kDa) after exposure to 0.2 μM Dox with or without pre-incubation with 40 μg/ml of the hAFS-CM_Hypo_ or with hNCTC-CM_Hypo_. Full-length blots are represented in Supplementary Fig. S4. (**d**) Densitometry of the western blot for p16^INK4a^ with same conditions as in (**c**). (**e**) Representative images of hCPC stained for cleaved-caspase-3 (Caspase-3): untreated cells (*Ctrl*), cells exposed to 1 μM Dox (*Dox*), and cells incubated with 40 μg/ml of the hAFS-CM_Hypo_ (*hAFS-CM*_*Hypo*_ + *Dox*) or with the hNCTC-CM_Hypo_ (*hNCTC-CM*_*Normo*_ + *Dox*) prior to Dox treatment. Scale bar 100 μm. (**f**) Percentage of hCPC expressing cleaved caspase-3 (% Caspase-3^+^ cells, mean ± s.e.m.). Ctrl: 0.90 ± 0.1%, Dox: 4.5 ± 0.6%, hAFS-CM_Hypo_ + Dox: 3.8 ± 0.5%, and hNCTC-CM_Hypo_ + Dox: 6.4 ± 1.5%.

Damage, senescence and depletion of human endogenous cardiac progenitor cells (hCPC) are thought to be critical for the pathogenesis of Dox cardiomyopathy^4^,^22^. Therefore, we assessed whether hAFS-CM_Hypo_ may also protect hCPC against Dox. hCPC were isolated from human atrial samples as previously described^23^ and treated with Dox with or without prior incubation with hAFS-CM_Hypo_ or hNCTC-CM_Hypo_. The frequency of SA β-galactosidase positive cells was significantly increased by Dox, as expected, and reduced by 51% by pre-incubation with hAFS-CM_Hypo_ (50.1 ± 6.4% vs 24.5 ± 1.9% of SA β-galactosidase positive cells), whilst hNCTC-CM_Hypo_ was not effective (Fig. 6a,b). The same trend was observed when senescence was evaluated by analysing the expression of p16^INK4a^, with hAFS-CM_Hypo_ decreasing p16^INK4a^ levels by 30% (2.3-fold increase vs 1.6-fold increase compared to untreated cells, Fig. 6c,d). In agreement with the results of previous, similar *in vitro* studies^4^,^24^, Dox induced hCPC apoptosis, as assessed by staining for activated caspase-3, to a limited extent (4.5 ± 0.6% vs 0.9 ± 0.1% cleaved caspase-3 positive cells, Fig. 6e,f). The rate of apoptosis was even lower when cells were pre-incubated with hAFS-CM_Hypo_ before exposure to Dox, although not significantly (4.5 ± 0.6% vs 3.8 ± 0.5% cleaved caspase-3 positive cells with Dox alone vs hAFS-CM_Hypo_ + Dox, respectively; Fig. 6e,f). No significant effects on either senescence or apoptosis markers were exerted by hAFS-CM or hNCTC-CM alone (see Supplementary Fig. S2).

## Discussion

This work provides robust evidence, gathered from the study of different cellular models, that the hAFS secretome (hAFS-CM) antagonizes Dox cardiotoxicity. As Dox-induced left ventricular dysfunction and heart failure are relatively common and by themselves have clinical burden, cause hospitalization, and carry a risk of mortality^1^,^5^, our results hold high translational potential value and great promise for future therapeutic strategies.

DNA damage has been pinpointed as the driver of the noxious effects of Dox on cardiomyocytes and CPC^10^, which eventually leads to their senescence and apoptosis^6^,^8^,^9^,^17^. These cellular alterations are the basis for a cardiomyopathy that typically has delayed clinical presentation. Although the cardiac cytotoxicity of Dox can be observed shortly after first infusion of the drug^25^, myocardial tissue derangement, cardiac dilatation and decline in left ventricular ejection fraction tend to occur towards the end of chemotherapy or thereafter - even years later^26^,^27^. Whereas depletion of terminally-differentiated cardiomyocytes is intuitively associated with decreased ventricular mass and, thereby, contractile force, senescence of CPC may worsen Dox cardiomyopathy by hindering the intrinsic ability of the myocardium to self-repair and regenerate via the activation of endogenous cardiac progenitors^22^. Previously published work showed that senescence is more pronounced than apoptosis in CPC exposed to Dox and may be primarily responsible for deficient CPC activity, conferring greater susceptibility to myocardial injury and, eventually, development of anthracycline-related cardiomyopathy^4^,^24^. We confirmed that Dox strongly triggered CPC senescence and found that hAFS-CM counteracted it; conversely, Dox-induced apoptosis of CPC survival was limited and, possibly because of such a small magnitude of effect, it was non-significantly decreased by hAFS-CM. Furthermore, hAFS-CM antagonized Dox-initiated DNA damage, senescence, and apoptosis of cardiomyocytes. Thus, we conclude that hAFS-CM offsets fundamental and early aspects of Dox cardiotoxicity.

In the last decade, many authors have demonstrated that structural and functional improvements obtained after engraftment of stem cells into the heart can largely be attributable to secreted factors, collectively indicated as *secretome*, rather than to the direct trans-differentiation of the exogenous stem cells into cardiovascular cells forming new viable tissue^28^,^29^,^30^. As a consequence, a current paradigm in cardiac regenerative medicine is to exploit the stem cell secretome for therapy. In a rat model of ischemia/reperfusion injury, we previously showed that systemic injection of hAFS-CM remarkably reduced infarct size in the short term to the same extent as administration of hAFS^14^. The experiments presented here integrate these earlier findings and confirm that, in general, the hAFS secretome is a valuable source of cardioprotective factors.

Other authors have reported that both transplantation of embryonic stem cells (ES) and the ES-CM oppose Dox-induced cardiomyopathy in mice^31^,^32^,^33^,^34^. Protection against Dox cardiotoxicity via potent paracrine effects has also been described for the secretome of mesenchymal stem cells (MSC) derived from either human induced pluripotent stem cells (iPS) or from bone marrow^35^. Overall, these results are consistent with ours and suggest that, in principle, the stem cell secretome may be employed to treat Dox cardiac side effects, while avoiding the problems related to standard cell therapy, such as immune rejection and teratogenicity. Yet, important limitations with ES and iPS or bone marrow MSC should not be overlooked: ES are not free of ethical constraints, and culture of iPS is technically challenging and rather time-consuming. These drawbacks are not encountered with adult bone marrow MSC, which, however, are obtained by invasive sampling with low yield and present limited self-renewal potential. By contrast, there are no ethical concerns related to hAFS, as they are isolated from remaining samples of amniotic fluid collected by amniocentesis for prenatal screening. Moreover, hAFS are endowed with remarkable self-renewal capability, ES-like properties - such as proliferative ability and expression of pluripotent markers - and a higher paracrine potential than that of adult stem cells. That hAFS are immature foetal cells and developmentally very “young”, may go some way to explain their more powerful paracrine potential than adult stem cells. Finally, they withstand cryopreservation for a long time while maintaining a stable karyotype^11^, which makes their banking and scale up expansion very feasible.

Multiple pathways may mediate the antagonism of Dox toxicity by the hAFS-CM. Here it is suggested a PI3K/Akt-dependent role for NF-κB and its target genes, *Il6* and *Cxcl1*. Direct evidence of the involvement of IL-6 and, secondarily, CXCL-1 was obtained by using specific blocking antibodies. IL-6 is released by stressed cardiomyocytes and promotes cardiac cell survival in an autocrine/paracrine fashion^18^. It acts through several intracellular mediators, including PI3K/Akt^36^,^37^, which raises the possibility of a feed-forward signalling loop, whereby PI3K/Akt promote the transcription of *Il6* and IL-6 activates PI3K/Akt. Furthermore, CXCL1, the murine homologue of IL-8, is an established angiogeneic factor^38^,^39^ and has been reported to exert anti-inflammatory and pro-survival effects in a mouse model of autoimmune myocarditis^19^. Interestingly, PI3K/Akt activity has repeatedly been linked to protection against Dox cardiotoxicity^16^,^40^, including by the ES-CM^32^. In addition, NF-κB is the nexus of one of the functional networks associated with the top 20 factors preferentially overexpressed in the iPS-derived MSC-CM, which has also been found to reduce Dox cardiotoxicity^35^. Hence, the secretomes of different stem cell types may have in common at least part of the cellular mechanisms by which they counteract Dox cardiac damage. As discussed above, however, compared with the conditioned medium of other stem cells, the hAFS-CM has unique characteristics, such as the ease of isolation from discarded clinical samples and the high-self renewal capacity in culture, making this cells an ideal source to exploit for future paracrine therapy.

The sensitivity of cardiac cells to Dox is dictated by the balance between drug retention and efflux, the latter being largely function of the activity of the transporter, ABCB1B (also known as Multidrug Resistance Protein 1 or P-glycoprotein). In cardiomyocytes, both induction of this gene by exogenous stimuli^41^, and overexpression via genetic construct^42^, confer resistance to Dox injury. Moreover, in patients there is a correlation between polymorphisms in ABCB1B and susceptibility to anthracycline cardiotoxicity^43^. We now report that the hAFS-CM also promotes *Abcb1b* transcription via PI3K/Akt and, as a consequence, Dox extrusion from cardiomyocytes. Remarkably, it has recently been demonstrated that NF-κB, which is recruited by the hAFS-CM, is a positive regulator of *Abcb1b* expression^20^. This work did not aim to evaluate the effects of hAFS-CM on cancer cells. Nonetheless, it is worth noting that in a human breast cancer cell line the hAFS-CM did not modulate *ABCB1* levels or Dox efflux, indicating that, at least in some tumour cell types, Dox metabolism is not enhanced by hAFS-CM. In terms of the prospect of a future therapeutic use of the hAFS secretome, this finding is reassuring.

We acknowledge that the present study has limitations. The data obtained with the embryonic H9c2 cardiomyoblasts and neonatal cardiomyocytes (mNVCM) were not replicated with mature adult cardiomyocytes. Since these cell types correspond to different stages of development and, thereby, display substantial phenotypic differences, this aspect may be especially relevant from a translational point of view and needs further evaluation. Furthermore, the effects of sustained incubation of cardiomyocytes and CPC with hAFS-CM need to be investigated, in order to define the optimal duration of treatment for possible therapeutic application. In addition, *in vivo* experiments are needed to investigate the effects of the hAFS-CM on mature adult cardiomyocytes and CPC *in vivo* while within their own microenvironment, which comprises other cell types possibly influenced by the hAFS-CM.

As far as the mechanisms of the hAFS-CM paracrine cardioprotection are concerned, it is likely that other signalling pathways, besides PI3K/Akt and a set of NF-κB responsive genes, contribute to the hAFS-CM prevention of Dox toxicity. Furthermore, detailed characterization of hAFS-CM composition is essential to pinpoint the soluble factors that specifically mediate the inhibition of Dox DNA damage, senescence, and apoptosis. Finally, a more detailed analysis of the possible influence of donor age on the hAFS secretome is required to understand whether there may be significant variability between cell batches obtained from different women. Indeed, in our study, we used the secretome of different hAFS obtained by pooling together amniotic fluid samples from 3 different donors in order to limit possible variations of donor age on cell proliferation.

In conclusion, we prove, in principle, that the hAFS secretome protects cardiomyocytes and CPC against Dox toxicity. These results substantiate the concept of a stem cell based paracrine approach via cell-free delivery of bioactive factors to prevent cardiotoxicity of Dox and possibly other anticancer agents. Such a treatment might be especially important for survivors of childhood cancer, in whom cardiac complications of chemotherapy can significantly curtail life expectancy. In addition, the data presented herein may lay the foundations for future research work, using the hAFS secretome as an easily obtainable and appealing source of paracrine cardioactive molecules that may become an *advanced medicinal product* for new cardiac regeneration strategies, without some of the ethical and technical limitations associated with embryonic and adult stem cell use.

## Methods

### Cell culture

hAFS were sorted for c-kit expression (CD117 MicroBead Kit, Miltenyi Biotechnology) within the cells isolated from left over samples of II trimester amniotic fluid, collected via prenatal screening amniocentesis, with informed consent from donors and proved negative for disease^11^,^44^,^45^. The protocol complied with the Helsinki Declaration and was approved by the local ethical committee Comitato Etico Regionale IRCCS AOU San Martino – IST (protocol 0036463/15 P.R. 428REG2015). Cells were cultured in Minimal Essential Medium (MEM) alpha with 15% fetal bovine serum (FBS), 1% L-glutamine, 1% penicillin/streptomycin, (Gibco-Thermo Fisher Scientific), 18% Chang B, and 2% Chang C (Irvine Scientific) at sub-confluence. To limit the intrinsic variability of primary cultures, which might be influenced by the donors different age (samples were obtained by women from 25 up to 46 years old, mean: 38.4 ± 3.3 years old), 3 different human amniotic fluid samples were pooled together to isolate hAFS for each experiment.

The NCTC 2544 cell line was purchased from the Interlab Cell Line Collection (Genova, Italy) and cultured in MEM/Earl’s Balanced Salt Solution (MEM/EBSS) with 10% FBS, 1% non-essential aminoacids, 1% L-glutamine, and 1% penicillin/streptomycin (all EuroClone).

The H9c2 cell line was bought from the European Collection of Authenticated Cell Cultures (Salisbury, UK) and cultured as already described^15^,^16^.

mNVCM were isolated via enzymatic digestion from 2-day-old C57/Bl6 mouse heart by multiple digestions in a collagenase II solution (300 U/ml, Worthington Biochemicals), according to^46^, and seeded on gelatin (1% solution, Sigma-Aldrich) coating at 10^5^ cells/cm^2^ in culture medium (69% Dulbecco′s Modified Eagle Medium, DMEM, 15% M199, 10% horse serum, 5% FBS, 1% penicillin/streptomycin and 1% L-glutammine, Gibco-Thermo Fisher Scientific). All animal procedures were carried out in compliance with national and international laws and specific authorisation (protocol 792/2015-PR from Animal Facility of IRCCS AOU San Martino-IST).

The MDA-MB-231 human breast cancer (adenocarcinoma) cell line was purchased from the American Type Culture Collection (ATCC, Virginia, US) and cultured in RPMI 1640 medium (Thermo Fisher) supplemented with 10% FBS, 1% non-essential aminoacids, 1% L-glutamine, and 1% penicillin/streptomycin (all EuroClone).

hCPC were isolated as previously described^23^ from human auricolae fragments, obtained following written informed consent from patients, in compliance with the Helsinki Declaration and upon approval of the local ethical committee IRCCS Istituto Europeo di Oncologia and Centro Cardiologico Monzino (protocol CCFM C9/607). Briefly, the myocardial tissue was repeatedly digested at 37 °C in a 3 mg/ml collagenase solution (Serva), cells were FACS-sorted (FACSAria, Beckton-Dickinson) for c-kit expression using an APC-conjugated antibody (anti-CD117, clone YB5.B8; BD Biosciences) and cultured in Ham’s F12 medium (Lonza) with 10% FBS (Thermo Fisher Scientific), 2 mM L-glutathione and 5 × 10^−3^ U/mL human erythropoietin (both Sigma-Aldrich), 10 ng/mL bFGF (Peprotech), and antibiotics (Lonza).

### Collection of hAFS-CM and hNCTC-CM

A schematic of hAFS-CM collection is shown in Supplementary Fig. S1. hAFS and NCTC 2544 cells were cultured for 24 hours in serum-free medium (SF: MEM alpha medium for hAFS and MEM/EBSS for NCTC 2544, both with 1% L-glutamine and 1% penicillin/streptomycin) in normoxia (20% O_2_ and 5% CO_2_ at 37 °C) or hypoxia (1% O_2_ and 5% CO_2_ at 37 °C in an hypoxic workstation, Baker Ruskinn, Carlibiotec s.r.l.). The hAFS-CM_Normo_, hAFS-CM_Hypo_, hNCTC-CM_Normo_ and hNCTC-CM_Hypo_ were concentrated 20 times using ultrafiltration membranes with a 3 kDa selective cut-off (Amicon Ultra-15, Millipore). Protein concentration was measured by Bradford assay. Samples were stored at −80 °C until use. At least 3 different batches of hAFS-CM and hNCTC-CM were used for each experiment.

### Experiment outline

hAFS-CM was used at 40 μg/ml, this concentration being the most protective in preliminary experiments with H9c2 cardiomyoblasts. The hNCTC-CM was employed at the same concentration. Cells were pre-incubated with hAFS-CM or hNCTC-CM for 3 hours prior to exposure to Dox. Apoptosis and cell viability were measured after Dox treatment (1 μM) for 21 hours. Blocking experiments were performed by adding 0.025  μg/ml anti-IL-6 and/or 0.25  μg/ml anti-CXCL1 antibodies (clone MP5-20F3 and 48415, respectively, R&D Systems) at the end of the 3 hour-incubation with the hAFS-CM when Dox treatment began, or 9 hours after hAFS-CM incubation, i.e. 6 hours after starting Dox treatment. Apoptosis was then evaluated 21 hours after Dox exposure. Senescence was examined after 3 hours of Dox exposure (H9c2: 0.1 μM, mNVCM and hCPC: 0.2 μM) followed by 42 hours in complete medium. DNA damage and gene expression profile were evaluated 6 hours after treatment with Dox (1 μM), whereas nuclear translocation of NF-κB was assessed after Dox (1 μM) incubation for 1.5 hours. To determine the acute effect on Akt phosphorylation, mNVCM were treated for 10 minutes with the hAFS-CM_Hypo_, with or without pre-treatment with LY-294002 (20 μM, Sigma-Aldrich) for 1 hour. Before every experiment, cells were incubated in low-serum culture medium (DMEM low-glucose with 0.5% FBS, 1% L-glutamine, and 1% penicillin-streptomycin) for 1 hour. Treatment outline is shown in Supplementary Fig. S1.

### Evaluation of senescence and apoptosis

To reveal SA β-galactosidase activity, cells were fixed in glutaraldehyde (0.5%) and incubated overnight at 37 °C in a solution containing citric acid (10 mM), potassium ferricyanide (5 mM), NaCl (150 mM), MgCl_2_ (2 mM), and 5-bromo-4-chloro-3-indolyl-beta-d-galactopyranoside (1 mg/ml, all Sigma Aldrich)^47^. Senescence of mNVCM was also revealed by immunostaining using a primary rabbit polyclonal antibody against p16^INK4a^ (Proteintech), followed by Vectastain secondary antibody, horseradish peroxidase (HRP)-streptavidin, and 3,3′-diaminobenzidine (all from Vector Laboratories) as detection system. Apoptosis was assessed by immunocytochemistry, using a rabbit monoclonal anti-cleaved caspase-3 antibody (clone 5A1E, Cell Signaling Technologies). The percentage of SA β-galactosidase, p16^INK4a^ and cleaved caspase-3 positive cells was evaluated considering six random fields (20x), taken with the Leica Q500 MC Image Analysis System (Leica).

### Cell viability assay

Following Dox exposure with or withour hAFS-CM or hNCTC-CM pre-incubation, MTT solution (250 μg/ml; Sigma-Aldrich) was added to the cells for 4 hours and absorbance read at 570 nm on a VersaMax (GE Intelligent Platforms) plate reader.

### Assessment of DNA damage

After exposure to Dox (1 μM) with or without pre-treatment with the hAFS-CM_Hypo_, mNVCM were fixed in paraformaldehyde solution (4%, Sigma Aldrich) and incubated with a rabbit monoclonal antibody against γH2aX (Ser139, clone 20E3, Cell Signalling Technologies) followed by Alexa Fluor488-conjugated secondary antibody (Thermo Fisher Scientific). Fluorescence emission was quantified using an Infinite 200 PRO plate reader (Tecan). Immunocytochemistry, immunofluorescence and cell culture images were acquired with an Axiovert microscope equipped with Axiovision software (Carl Zeiss).

### Gene expression profiling

The gene expression profile of mNVCM treated with Dox (1 μM) with or without pre-incubation with the hAFS-CM_Hypo_, was analysed by Affymetrix MG 430.2 microarray technology. Total RNA was extracted using the RNeasy Micro Kit (Qiagen). cRNA for hybridization was prepared using the GeneChip^®^ 3′ IVT PLUS Reagent Kit following the instructions provided by Affymetrix. Hybridisation and scanning were performed by standard procedures, as recommended by Affymetrix. Data were deposited in the Gene Expression Omnibus repository ( www.ncbi.nlm.nih.gov/geo/, accession number: GSE74513) and pre-processed using the RMA algorithm with quantile normalization^48^. Differentially expressed genes were identified applying a threshold of two-fold increase or decrease of the expression values when comparing the two conditions. Enrichment of functional categories of genes was analyzed by EnrichR Gene Set Enrichment Analysis ( http://amp.pharm.mssm.edu/Enrichr/)^49^.

### Real-time quantitative RT-PCR analysis

Total RNA from mNVCM and MDA-MB-231 cells was extracted using Qiazol Lysis Reagent (Qiagen) and cDNA obtained using the iScriptTM cDNA Syntesis Kit (Bio-Rad). Real-time qRT-PCR was carried out on a 7500 Fast Real-Time PCR System (Thermo Fisher Scientific) using Syber Green Master Mix (Thermo Fisher Scientific). Primer sequences for mouse *Il6*, *Cxcl1*, *Abcb1b*, *Hprt,* and human *ABCB1* and *GAPDH* were designed using the NCBI Primer-Blast tool ( http://www.ncbi.nlm.nih.gov/tools/primer-blast/; see Supplementary Table S2 for sequences). Gene expression levels were normalized using *Hprt* or *GAPDH* as endogenous control by applying the 2^−ddCt^ method.

### Western blot

Whole cell lysates were separated on a tris-glycine gel (8–16%, Thermo Fisher Scientific), transferred onto a poly-vinylidene difluoride membrane (Bio-Rad), and incubated overnight with the following primary antibodies: anti-phosphorylated Akt (Ser^473^ rabbit monoclonal, clone D9E; Cell Signaling Technologies); anti-Akt (rabbit polyclonal, cone 11e7 Cell Signalling Technologies); anti-phosphorylated IκBα (Ser^32/36^ mouse monoclonal, clone 5A5, Cell Signalling Technologies); anti total-IκBα (mouse monoclonal, clone 112B2, Cell Signalling Technologies); and anti-p16^INK4a^ (rabbit polyclonal, Proteintech). The membrane was incubated with goat anti-rabbit or anti-mouse HRP-conjugated secondary antibodies (sc-2030 and sc-2005, respectively; Santa Cruz Biotechnology). Bands were detected by Clarity Western ECL Substrate (Bio-Rad), imaged using Alliance LD2 system and software (UVItec), and quantified by densitometry analysis with ImageJ software (NCBI). Values were normalized against those of GAPDH, as revealed by a primary antibody directly conjugated to HRP (clone D16H11, Cell Signalling Technologies).

### Quantification of nuclear levels of NF-κB

Cells were first lysed in an hypotonic solution containing Tris-HCl (20 mM), NaCl (10 mM), MgCl_2_ (3 mM), PMSF (1 mM), and 1x PIC (all Thermo Fisher Scientific) in order to separate the cytoplasmic and nuclear fractions. Nuclear proteins were then retrieved by disrupting the nuclear fraction in an extraction buffer (1 mM Tris pH 7.4, 2 mM Na_3_VO_4_, 100 mM NaCl, 1 mM EDTA, 10% glycerol, 1 mM NaF, 0.5% deoxycholate, 20 mM Na_4_P_2_O_7_, and 0,5% NP40), and quantified by means of Bio-Rad protein assay (Bio-Rad). Levels of nuclear p65 were assessed using a NF-κB p65 ELISA kit (Thermo Fisher Scientific).

### Measurement of Dox fluorescence in culture medium

mNVCM and MDA-MB-231 cells were treated as described above. Their conditioned medium was collected at 0.5, 2, 4, 8, 16 and 24 h after Dox administration and immediately evaluated for fluorescence emission on a Infinite reader M200 (Tecan) equipped with a 530/25 nm excitation and a 590/20 nm emission filter (Dox excitation/emission peak).

### Statistical analyses

Results are presented as mean ± s.e.m. of at least three (n = 3) independent replicated experiments. Comparisons were drawn by two-way ANOVA (fluorescence intensity of the mNVCM- and MDA-MB-231-conditioned medium after exposure to Dox with or without pre-incubation with the hAFS-CM_Hypo_) or one-way ANOVA followed by post-hoc Tukey’s multiple comparisons test (all other data). Statistical analysis was performed using GraphPad Prism Version 6.0a (GraphPad Software) with statistical significance set at p < 0.05.

## Additional Information

**Accession codes:** Data deposited in the Gene Expression Omnibus repository are available at: www.ncbi.nlm.nih.gov/geo/, accession number: GSE74513.

**How to cite this article**: Lazzarini, E. *et al*. The human amniotic fluid stem cell secretome effectively counteracts doxorubicin-induced cardiotoxicity. *Sci. Rep.* **6**, 29994; doi: 10.1038/srep29994 (2016).

## Supplementary Material

Supplementary Information
