# Supplementary material for: The human amniotic fluid stem cell secretome effectively counteracts doxorubicin-induced cardiotoxicity
Source: Sci Rep. 2016 Jul 21;6:29994. doi: 10.1038/srep29994 (PMC4956770; doi:10.1038/srep29994)
Supplement: Supplementary Information [file srep29994-s1.doc]

**Supplementary Information**

**The human amniotic fluid stem cell secretome effectively counteracts doxorubicin-induced cardiotoxicity**

Edoardo Lazzarini^1,§^, Carolina Balbi^2,§^, Paola Altieri^1^, Ulrich Pfeffer^3^, Elisa Gambini^4^, Marco Canepa^1^, Luigi Varesio^5^, Maria Carla Bosco^5^, Domenico Coviello^6^, Giulio Pompilio^4^, Claudio Brunelli^1^, Ranieri Cancedda^2^, Pietro Ameri^1#*^, and Sveva Bollini^2#*^.

^1^ Cardiovascular Biology Laboratory, Department of Internal Medicine, University of Genova, Genova, 16132, Italy.

^2^ Regenerative Medicine Laboratory, Department of Experimental Medicine, University of Genova, Genova, 16132, Italy.

^3^ Molecular Pathology Unit, IRCCS AOU San Martino-IST, Istituto Nazionale per la Ricerca sul Cancro, Genova, 16132, Italy.

^4^ Vascular Biology and Regenerative Medicine Unit, IRCCS Centro Cardiologico Monzino, Milano, 20138, Italy.

^5^ Molecular Biology Laboratory, IRCCS Istituto Giannina Gaslini, Genova, 16147, Italy.

^6^ Human Genetics Laboratory, E.O. Ospedali Galliera, Genova, 16128, Italy.

* Correspondence to: [sveva.bollini@unige.it](mailto:sveva.bollini@unige.it) or [pietroameri@unige.it](mailto:pietroameri@unige.it)

Work telephone number: +390105558393.

*§ Joint first authorship.*

*# These authors contributed equally to the work.*

**Supplementary Figure 1**

**
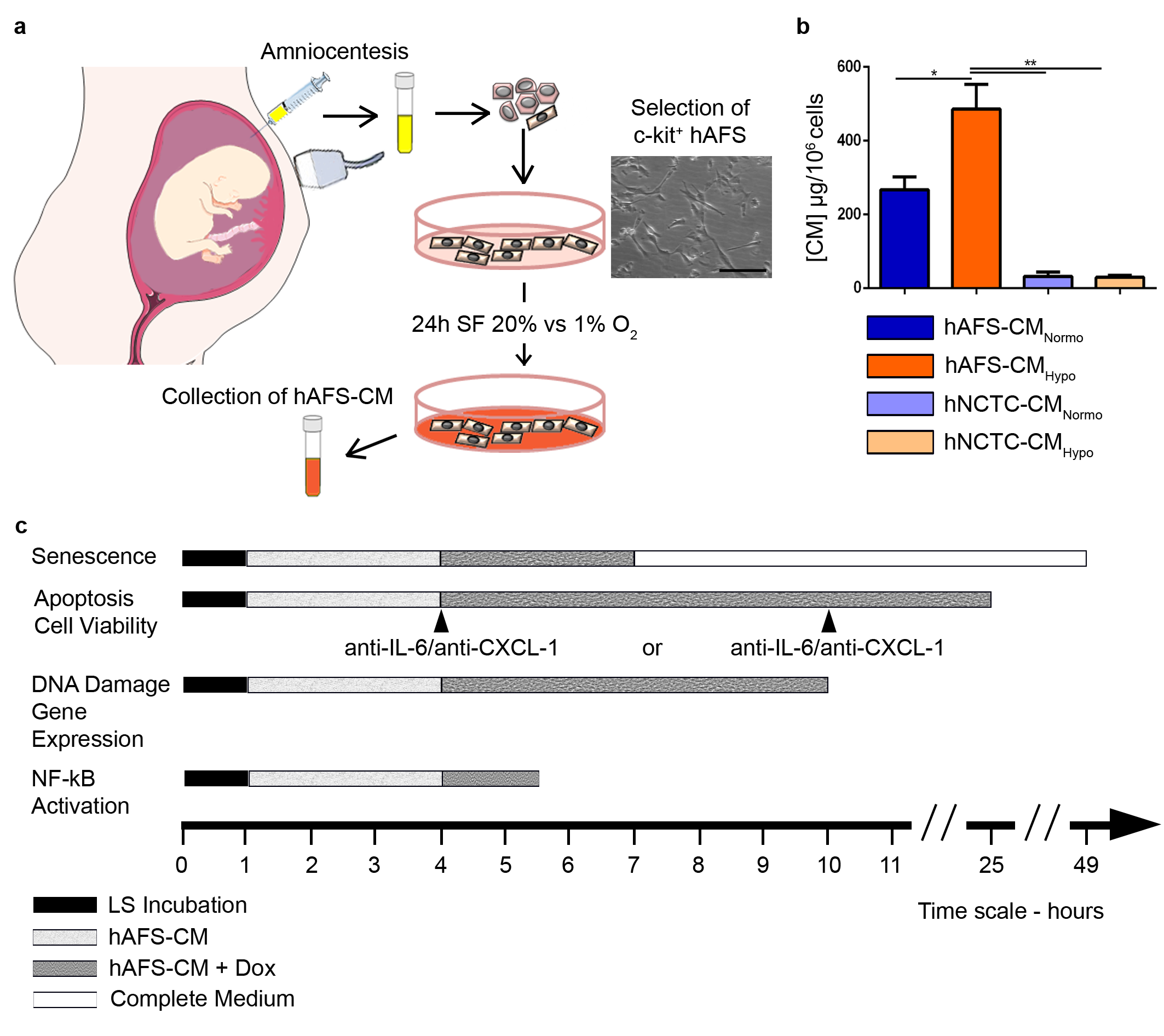
**

**Supplementary Fig. S1. Schematic of the hAFS secretome isolation and of experimental design.**

**a)** Schematic illustration of the isolation of hAFS and of hAFS-CM from left over samples of II trimester amniotic fluid obtained via amniocentesis for prenatal screening. Scale bar 100μm. *SF*: serum free medium (part of the illustration has been modified and produced using and with permission, of Servier Medical Art, [*www.servier.com*](http://www.servier.com/)). **b**) Bradford assay to determine protein concentration in hAFS-CM_Normo_ (266.6±35.2 μg/10^6^ cells), hAFS-CM_Hypo_ (485.1±67.1 μg/10^6^ cells), hNCTC-CM_Normo_ (31.6±12.4 μg/10^6^ cells) and hNCTC-CM_Hypo_ (29.4±5.1 μg/10^6^ cells). * p<0.05 (p=0.0172), ** p<0.01 (p=0.0037), *CM*: Conditioned Medium. **c**) Schematic representation of the treatment outline; *LS*: Low Serum (0.5% FBS) culture medium, *Complete Medium*: Complete Culture Medium with FBS; *anti-IL-6/anti-CXCL-1*: blocking antibody anti-IL-6 or anti-CXCL-1.

**Supplementary Figure 2**

**
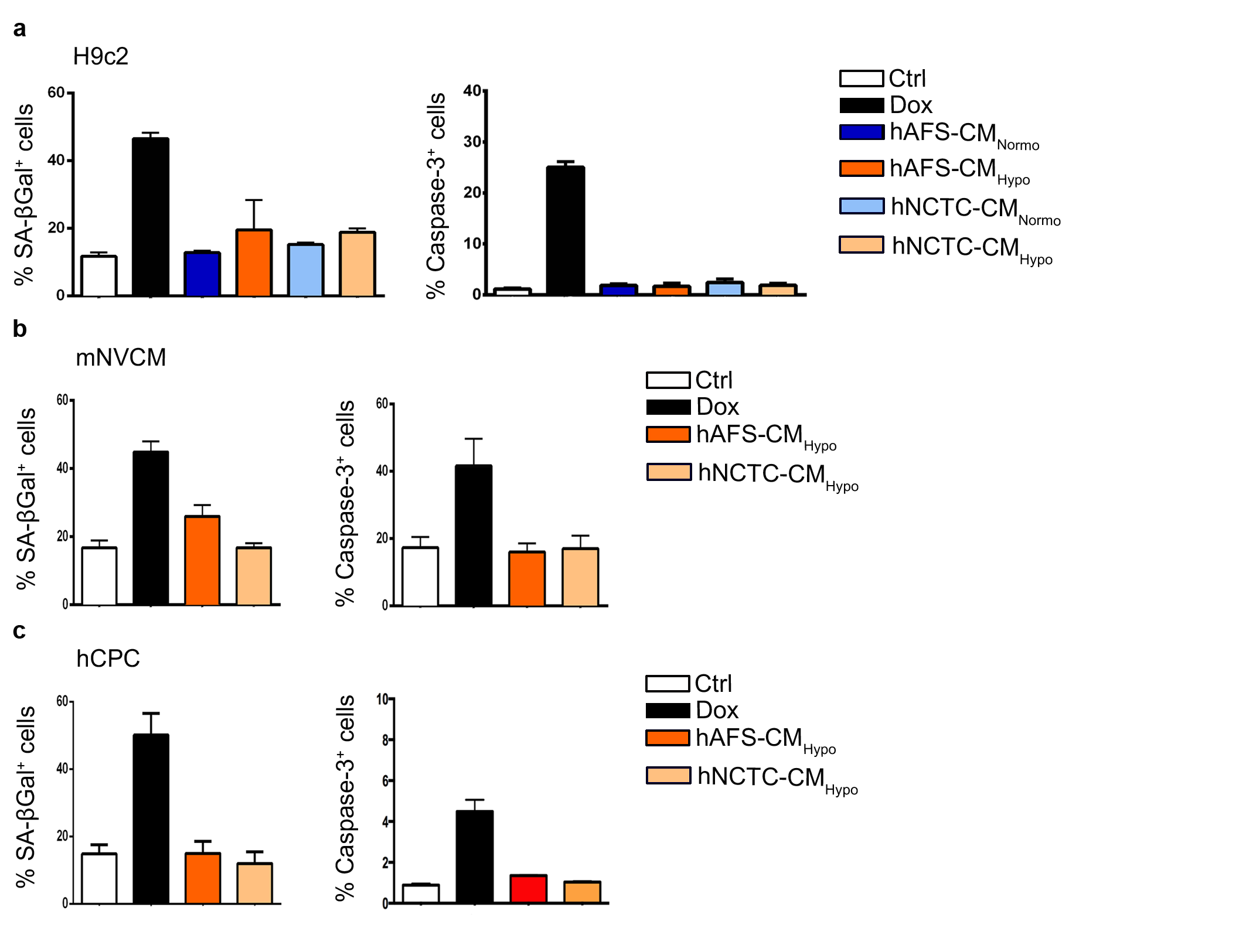
**

**Supplementary Fig. S2. Treatment with hAFS-CM or hNCTC-CM alone does not cause senescence or apoptosis of H9c2 cells, mNVCM or hCPC.**

Percentage of H9c2 cells (**a**), mNVCM (**b**), and hCPC (**c**) positive for senescence associated (SA) β-galactosidase or cleaved caspase-3 cells (% Caspase-3^+^ cells) following no treatment (*Ctrl*), exposure to Dox (*Dox*), or incubation with 40μg/ml of hAFS-CM_Normo_, hAFS-CM_Hypo_, hNCTC-CM_Normo_, or hNCTC-CM_Hypo_ alone. To induce senescence, Dox was used at 0.1μM (H9c2) or 0.2μM (mNVCM and hCPC), whereas apoptosis was triggered by exposing all cell types to 1μM Dox.

**Supplementary Figure 3**


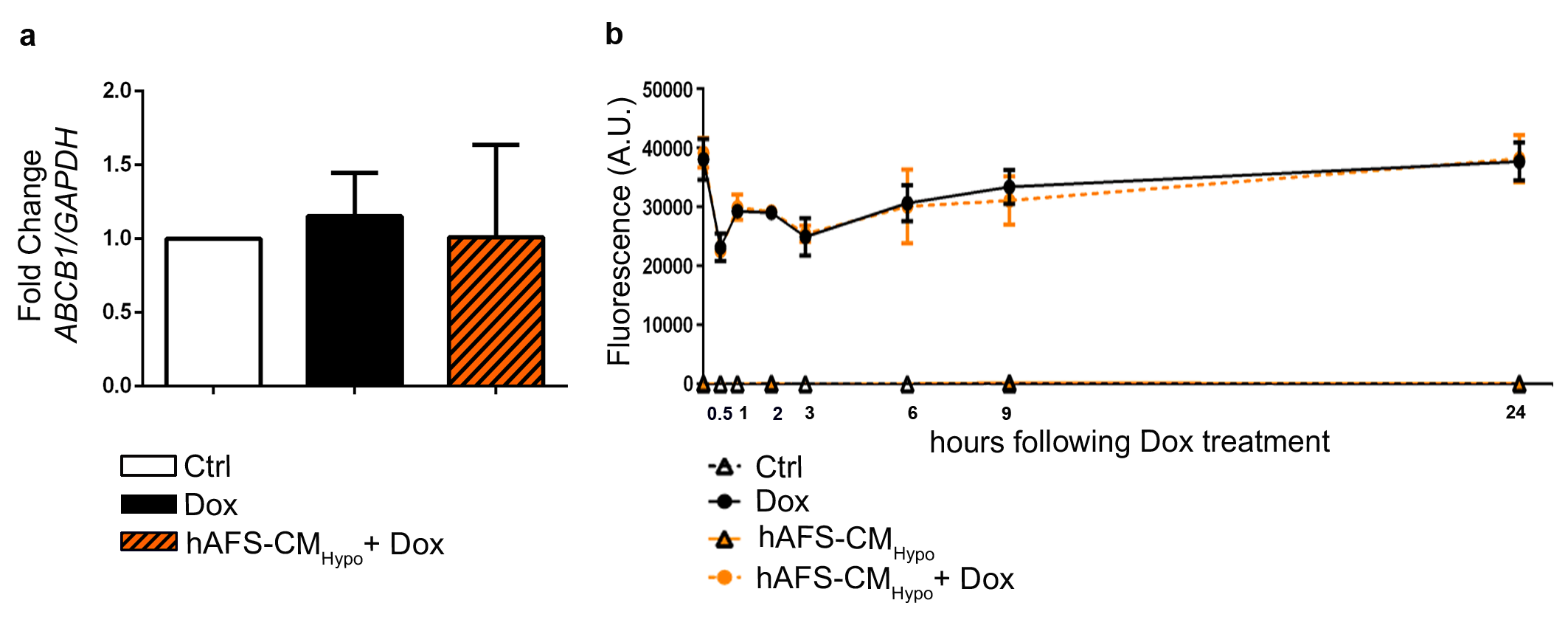


**Supplementary Fig. S3 The hAFS-CM_Hypo_ doesn’t modulate *Abcb1b* expression or the efflux of Dox from the human breast cancer MDA-MB-231 cells.**

**a**) Real time qRT-PCR showing no significant modulation of the *ABCB1* gene in the human breast cancer MDA-MB-231 cells incubated with 40μg/ml hAFS-CM_Hypo_ before exposure to 1μM Dox (*hAFS-CM_Hypo_ + Dox*), compared to untreated cells (*Ctrl*) or cells exposed to Dox (*Dox*); **b**) Quantification of Dox fluorescence in MDA-MB-231-conditioned medium at 0.5, 1, 2, 3, 6, 9 and 24h following incubation with 40ug/ml hAFS-CM_Hypo_ and/or exposure to 1μM Dox. *A.U.*: Arbitrary Unit.

**Supplementary Figure 4**

**
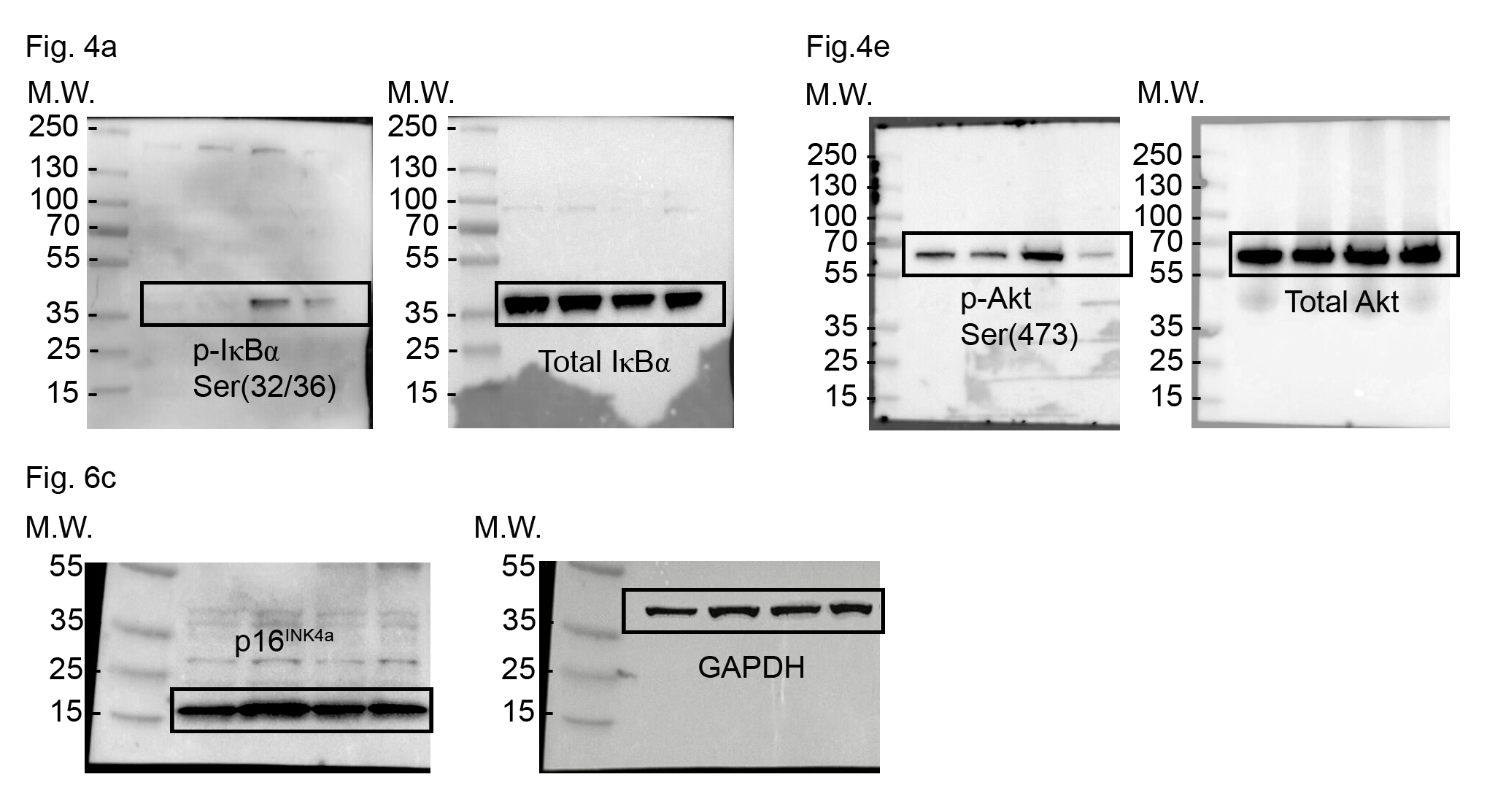
**

**Supplementary Fig. S4. Uncropped western blot scans with size marker indications.**  Uncropped, full-length blots referring to panels used in Fig. 4a, Fig. 4e and Fig. 6c of the manuscript (the bands shown are indicated in the box). Images were acquired using Alliance LD2 system and software (UVItec).

| ***EnrichR Gene Set Enrichment Analysis - Gene Ontology: Biological Function*** | | | |
| --- | --- | --- | --- |
| Term | GO-ID | p value | Genes |
| **Cytokine activity** | GO:0005125 | **5.2E-04** | ***Il6****, Ccl2,* ***Cxcl1****, Tnfrsf11b, Timp1, Cxcl3, Inha, Cxcl2, Areg, Cxcl5* |
| **Chemokine activity** | GO:0008009 | **2.9E-03** | *Ccl2,* ***Cxcl1****, Cxcl3, Cxcl2, Cxcl5* |
| Oxidoreductase activity, oxidizing metal ions | GO:0016722 | 7.6E-03 | *Steap4, Cyb561, Cp* |
| **Chemokine receptor binding** | GO:0042379 | **4.6E-03** | *Ccl2,* ***Cxcl1****, Cxcl3, Cxcl2, Cxcl5* |
| **Growth factor activity** | GO:0008083 | **7.6E-03** | ***Il6,*** *Timp1, Inha, Areg, Thbs4, Pgf* |
| Cell adhesion molecule binding | GO:0050839 | 7.6E-03 | *Vcam1, Actn1, Itgb2, Adam22, Tgfbi, Pvr, Thbs4* |
| **Cytokine receptor binding** | GO:0005126 | **2.4E-02** | *Socs2,* ***Il6****, Ccl2,* ***Cxcl1****, Cxcl3, Cxcl2, Cxcl5* |
| Integrin binding | GO:0005178 | 2.4E-02 | *Vcam1, Actn1, Adam22, Tgfbi, Thbs4* |
| Collagen binding | GO:0005518 | 2.4E-02 | *Lum, Abi3bp, Tll1, Tgfbi* |
| Cargo receptor activity | GO:0038024 | 2.4E-02 | *Scara3, Olr1, Enpp3, Lrp8* |

**Supplementary Table S1. Analysis of the genes most significantly up-regulated by mNVCM following incubation with hAFS-CM and treatment with Dox. *GO-ID*: Gene Ontology Identification code; highlighted in bold the genes analysed in this study.**

| Symbol | NCBI  Gene ID | Sequence | Length (bp) |
| --- | --- | --- | --- |
| *mIl6* | 16193 | Forward:  5′-TCGTGGAAATGAGAAAAGAGTTGTG-3’  Reverse:  5’-CCAGTTTGGTAGCATCCATCATTT-3’ | 124 |
| *mCxcl1* | 14825 | Forward:  5’-ACTTGGGGACACCTTTTAGCA-3’  Reverse:  5′-ACTCAAGAATGGTCGCGAGG-3′ | 89 |
| *mAbcb1b* | 18669 | Forward:  5′-AAACTCCATCACCACCTCACG-3′  Reverse:  5′-ATCTTCTGAGGTTCCGCTCAA-3′ | 104 |
| *mHprt*  *hGAPDH*  *hABCB1* | 15452  2597  5243 | Forward:  5′-CCCCAAAATGGTTAAGGTTGC-3′  Reverse:  5′-CCAACAAAGTCTGGCCTGTAT-3′  Forward:  5’-ATGGCACCGTCAAGGCTGAGAA-3’  Reverse:  5’-CCAGCATCGCCCCACTTGATT -3’  Forward:  5’-GGAGGCCAACATACATGCCT-3’  Reverse:  5’-AGGCTGTCTAACAAGGGCAC-3’ | 78  103  133 |

**Supplementary Table S2. Primer sequences used in the qRT-PCR analysis.** *mIl6*, *mCxcl1*, *mAbcb1b* and *mHprt*: mouse *Il6*, mouse *Cxcl1*, mouse *Abcb1b* and mouse *Hprt*; *hGAPDH*, and *hABCB1*: human *GAPDH* and human *ABCB1*.
